# One Result of Koch’s Investigations

**Published:** 1883-04

**Authors:** 


### ONE RESULT OF KOCH'S INVESTIGATIONS.

In a letter to the *Philadelphia Medical Times*, Dr. Formad states that by a German Imperial order, phthisical patients in the military hospitals are as carefully isolated as though they had small-pox, and infection is as sedulously guarded against. The disease is regarded as eminently contagious, and consumptives are avoided. In this country we already begin to see the effects of belief in a specific tuberculous contagion. Instances are on record where people suffering from phthisis have been shunned, and even neglected by their friends, through fear of innoculation. The moral effects of such a social ostracism cannot but exert an evil influence upon an invalid. When a person afflicted with one of the most common disorders of our day becomes an object of dread, instead of active sympathy, the horrors of phthisis are increased a hundred fold.

